# Identification of gene expression patterns crucially involved in experimental autoimmune encephalomyelitis and multiple sclerosis

**DOI:** 10.1242/dmm.025536

**Published:** 2016-10-01

**Authors:** Martin M. Herrmann, Silvia Barth, Bernhard Greve, Kathrin M. Schumann, Andrea Bartels, Robert Weissert

**Affiliations:** 1 Department of General Neurology, Hertie Institute for Clinical Brain Research, University of Tübingen, 72076 Tübingen, Germany; 2 Department of Neurology, University of Regensburg, 93053 Regensburg, Germany

**Keywords:** Experimental autoimmune encephalomyelitis, Multiple sclerosis, Central nervous system, Cellular traffic, T cell, Adjuvant

## Abstract

After encounter with a central nervous system (CNS)-derived autoantigen, lymphocytes leave the lymph nodes and enter the CNS. This event leads only rarely to subsequent tissue damage. Genes relevant to CNS pathology after cell infiltration are largely undefined. Myelin-oligodendrocyte-glycoprotein (MOG)-induced experimental autoimmune encephalomyelitis (EAE) is an animal model of multiple sclerosis (MS), a chronic autoimmune disease of the CNS that results in disability. To assess genes that are involved in encephalitogenicity and subsequent tissue damage mediated by CNS-infiltrating cells, we performed a DNA microarray analysis from cells derived from lymph nodes and eluted from CNS in LEW.1AV1 (*RT1^av1^*) rats immunized with MOG 91-108. The data was compared to immunizations with adjuvant alone or naive rats and to immunizations with the immunogenic but not encephalitogenic MOG 73-90 peptide. Here, we show involvement of *Cd38*, *Cxcr4* and *Akt* and confirm these findings by the use of *Cd38*-knockout (B6.129P2-*Cd38^tm1Lnd^*/J) mice, S1P-receptor modulation during EAE and quantitative expression analysis in individuals with MS. The hereby-defined underlying pathways indicate cellular activation and migration pathways mediated by G-protein-coupled receptors as crucial events in CNS tissue damage. These pathways can be further explored for novel therapeutic interventions.

## Abstract (precis)

**Summary:** To define disease-inducing immune responses, unbiased gene expression in LEW.1AV1 (*RT1^av1^*) rats immunized with autoantigen-derived encephalitogenic and non-encephalitogenic peptides was investigated in MOG-EAE. CXCR4, CXCL12, AKT and CD38 control CNS inflammation.

## INTRODUCTION

Multiple sclerosis (MS) is a disease of the central nervous system (CNS) that leads to chronic inflammation, demyelination, and axonal and neuronal loss, resulting in disability (Noseworthy et al., 2000; Weissert, 2013). Myelin-oligodendrocyte-glycoprotein (MOG)-induced experimental autoimmune encephalomyelitis (EAE) in rats reproduces major aspects of the human pathology (Weissert et al., 1998b; Storch et al., 1998; Kornek et al., 2000; Weissert, 2016). MOG is expressed on the outer surface of the myelin sheath. In contrast to merely T-cell-mediated animal models, the pathogenesis of MOG-induced EAE in the rat involves the combined action of T and B cells, antibodies and macrophages, mimicking type II lesions in MS (Genain et al., 1995; Mathey et al., 2004; Lucchinetti et al., 2000).

Encephalitogenic peptides presented on MHC class II molecules to T cells lead to a program that forces lymphocytes to be activated and migrate towards the CNS (Riedhammer and Weissert, 2015). Adjuvant contributes by affecting multiple signaling pathways in lymphocytes as well as in organ-resident cells like in the CNS. We have previously demonstrated that MOG 91-108 is the major determinant to trigger disease in rats expressing *RT1^av1^* or *RT1^n^* haplotypes (Weissert et al., 2001). Interestingly, the capacity of MOG 91-108 to induce EAE was dissociated in regard to Th1 or Th2 cytokine expression in lymphoid tissue compared to the CNS. Moreover, different MOG 1-125-derived peptides, such as MOG 73-90, were immunogenic, showing strong Th1 responses, but were not encephalitogenic. The induction of active EAE in LEW MHC congenic rat strains and Dark Agouti (DA) (*RT1^av1^*) rats does not require the application of pertussis toxin like in mice. This is an advantage because the exact role of pertussis toxin in EAE induction is not clear so far. Pertussis toxin inhibits Gi proteins and thereby influences multiple cellular processes and pathways (Dumas et al., 2014). Active EAE in susceptible rat strains is induced by immunization with an encephalitogenic peptide mixed with mineral oil [incomplete Freund's adjuvant (IFA)] with the addition of heat-inactivated mycobacterium tuberculosis (MT) as adjuvant [complete Freund's adjuvant (CFA)]. MT leads by binding and signaling through Toll-like receptors (TLRs) to an activation program in a number of cell types and is also a systemic ‘danger signal’ (Mills, 2011).

In regard to susceptibility to EAE and MS, gene expression profiling studies have been performed to elucidate genes that are involved in disease pathogenesis. A number of interesting genes were described, such as osteopontin (Hur et al., 2007). In no study was a systematic comparison of gene expression profiles performed in EAE in which the influence of adjuvant and antigen was systematically compared on the expression profile of lymph node (LN)-derived cells or cells eluted from CNS of diseased animals. In the present study, we systematically compared the gene expression profiles of cells from draining LNs and CNS-infiltrating cells that were eluted in LEW.1AV1 (*RT1^av1^*) rats immunized with MOG 91-108 in CFA, CFA alone and in naive rats. Moreover, we compared the gene expression profile of rats immunized with the encephalitogenic MOG peptide 91-108 with rats immunized with the non-encephalitogenic MOG peptide 73-90. We found differentially expressed genes that are of major importance for encephalitogenicity. The influence of these genes was subsequently verified by different means.

## RESULTS

### Gene expression after immunization with encephalitogenic and non-encephalitogenic peptides

One of the important issues in MS and other inflammatory diseases of the CNS is understanding the requisites of autoantigenic peptides to induce CNS inflammation (Riedhammer and Weissert, 2015). Beside presentation of autoantigen-derived peptides on MHC molecules and the availability of reactive T-cell and B-cell repertoires as well as the presence of the target antigen in the CNS, pathways of cellular activation exist that allow disease development. These pathways are presently only partly elucidated. We used MOG-induced EAE in LEW.1AV1 (*RT1^av1^*) rats as a model system for CNS inflammation. In this EAE model, the determinant MOG 91-108 is immunogenic and encephalitogenic. In contrast, the determinant MOG 73-90 is immunogenic but not encephalitogenic. We assessed the gene expression profiles by gene arrays of lymphocytes from draining LNs and from lymphocytes eluted from the CNS.

To focus on genes that are truly relevant to encephalitogenicity and not simply involved in general inflammatory responses, we compared gene arrays of LEW.1AV1 (*RT1^av1^*) rats immunized with the encephalitogenic MOG stretch MOG 91-108 to naive LEW.1AV1 (*RT1^av1^*) rats and rats immunized with the adjuvant CFA alone as well as rats immunized with the non-encephalitogenic MOG 73-90 determinant. We analyzed ten comparisons for each of the naive and CFA groups versus MOG 91-108 and five comparisons for MOG 73-90 versus MOG 91-108. The number of comparisons in which a given gene had a signal log ratio (SLR) of above 1 was counted. In Table 1, we show genes that are upregulated in at least half of the comparisons (=50%). Besides *Cxcr4* and *Cd38*, which were subsequently analyzed in greater detail, many genes with a known function in EAE and MS pathology were found to have an increased expression in MOG 91-108-immunized rats as compared to controls. This validates our gene list and supports the relevance of the genes not previously described in EAE. In Table S1, genes with decreased expression in EAE are listed. In this analysis, the variability between gene arrays was much higher and fewer genes were found to be regulated with a clear pattern according to our criteria.

**Table 1.**
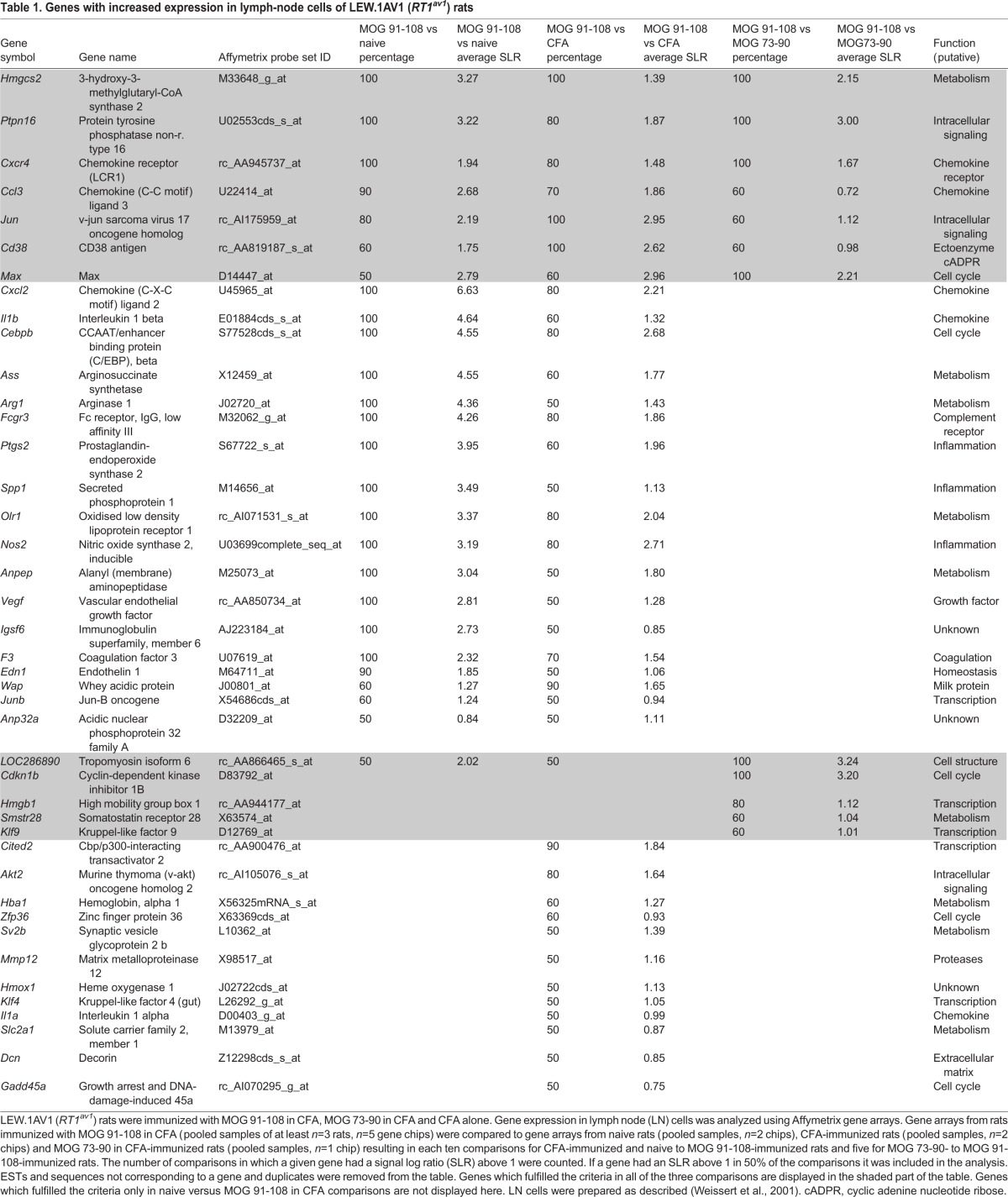
**Genes with increased expression in lymph-node cells of LEW.1AV1 (*RT1^av1^*) rats**

Comparisons of microarrays of CNS-infiltrating lymphocytes derived from LEW.1AV1 (*RT1^av1^*) rats after immunization with MOG 91-108, MOG 73-90 and CFA alone resulted in many more genes being differentially expressed as compared to the analysis of LN cells (Tables S2 and S3). This could mirror the influx of different cell populations into the CNS during an inflammatory attack. Similar to the analysis of LN cells, we found that *Cd38* and *Cxcr4* mRNA was strongly increased in CNS-infiltrating cells.

Subsequently, we analyzed purified CD4^+^ cells from LNs and CNS of MOG 91-108- and MOG 73-90-immunized rats. To some extent, similar gene expression profiles were found in the purified CD4^+^ cell population compared to non-separated LN cells (Table 1, Tables S4 and S5).

Owing to their strong expression in MOG 91-108-immunized rats compared to naive, CFA-immunized and MOG 73-90-immunized rats, we chose *Cxcr4* and *Cd38* for further analysis.

### *Cxcr4* and *Cd38* in EAE in LEW.1AV1 (*RT1^av1^*) rats

**Fig. 1.**
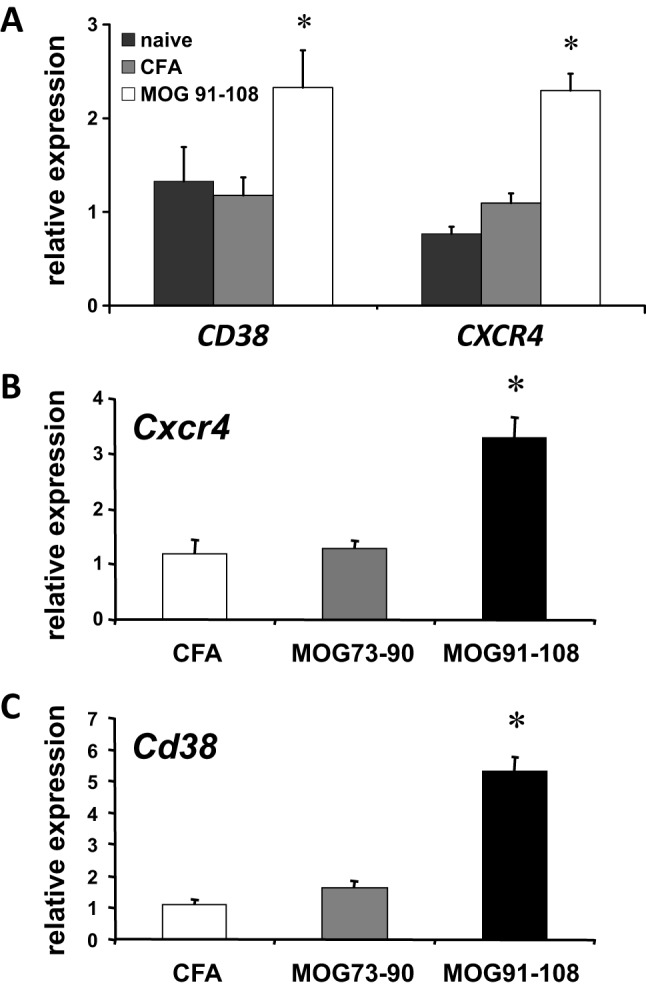
***Cxcr4* and *Cd38*** **expression in lymph node cells and CNS.** (A) Quantitative SYBR green real-time PCR was performed for *Cxcr4* and *Cd38* in lymph node (LN) cells from naive LEW.1AV1 (*RT1^av1^*) rats (black bars, *n*=6), or those immunized with CFA (gray bars, *n*=8) or MOG 91-108 in CFA (white bars, *n*=8), on day 12 p.i. Increased *Cxcr4* (ANOVA, *P<0.0001) and *Cd38* (ANOVA, *P<0.05) expression was found in MOG 91-108 in CFA- immunized rats compared to naive and CFA-alone-immunized rats. (B) Quantitative expression of *Cxcr4* in cells eluted from the CNS of CFA (white bars, *n*=6)-, MOG 73-90 in CFA (gray bars, *n*=6)- and MOG 91-108 in CFA (*n*=6)-immunized LEW.1AV1 (*RT1^av1^*) rats. *Cxcr4* was upregulated in MOG 91-108 in CFA-immunized rats compared to the other groups (ANOVA, *P<0.001) on day 12 p.i. (C) Quantitative expression of *Cd38* of lymphocytes eluted from the CNS of CFA (white bars, *n*=6)-, MOG 73-90 in CFA (gray bars, *n*=6)- and MOG 91-108 in CFA (*n*=6)-immunized LEW.1AV1 (*RT1^av1^*) rats on day 12 p.i. *Cd38* was upregulated in MOG 91-108 in CFA-immunized rats compared to the other groups (ANOVA, *P<0.001). Results are expressed as 2^−ΔΔ CT^ values. Numbers are mean±s.e.m.

Confirming our microarray results by quantitative PCR, we found a significant upregulation of *Cxcr4* expression in LN cells of MOG 91-108-immunized rats (*n*=8) as compared to CFA-immunized (*n*=8, ANOVA, *P*<0.0001) and naive (*n*=6, ANOVA, *P*<0.0001) LEW.1AV1 (*RT1^av1^*) rats (Fig. 1A). Also, an increased expression of *Cd38* was measured [MOG 91-108-immunized rats (*n*=8) as compared to CFA-immunized (*n*=8, ANOVA, *P*<0.05) and naive (*n*=6, ANOVA, *P*<0.05) LEW.1AV1 (*RT1^av1^*) rats].

In cells eluted from the CNS, we found upregulation of *Cxcr4* (Fig. 1B) and *Cd38* (Fig. 1C) in MOG 91-108-immunized LEW.1AV1 (*RT1^av1^*) rats (*n*=6) compared to rats immunized with MOG 73-90 (*n*=6, ANOVA, *Cxcr4* and *Cd38* each *P*<0.001) or CFA alone (*n*=6, ANOVA, *Cxcr4* and *Cd38* each *P*<0.001).

### *Cxcr4* and *Cxcl12* expression in spinal cord of DA (*RT1^av1^*) rats

**Fig. 2.**
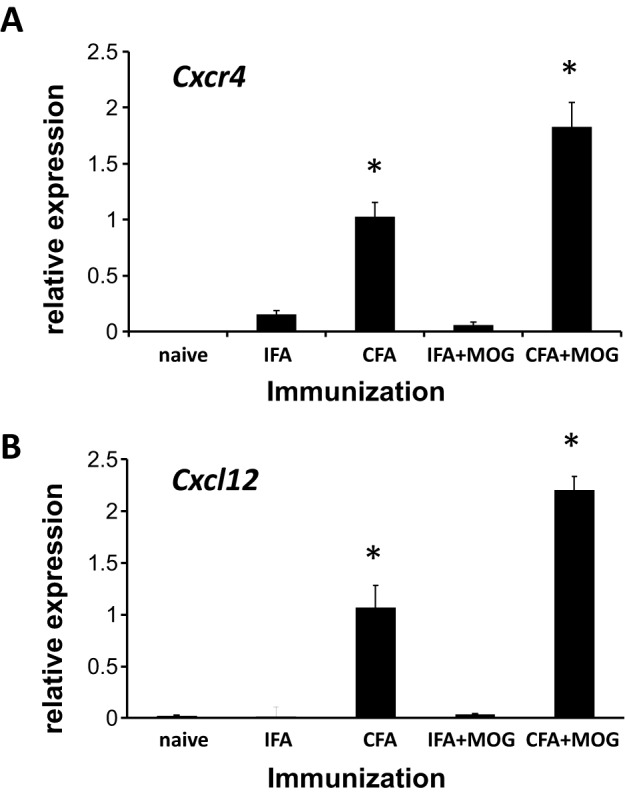
***Cxr4* and *Cxcl12* expression in spinal cord of DA (*RT1^av1^*) rats.** Quantitative SYBR green real-time PCR was performed for *Cxcr4* (A) and *Cxcl12* (B) from PBS-perfused spinal cord tissue of naive DA (*RT1^av1^*) rats (*n*=4), rats immunized with IFA (*n*=4) or CFA alone (*n*=4) and DA rats immunized with MOG 1-125 in IFA (*n*=4) or MOG 1-125 in CFA (*n*=4) on day 12 p.i. Increased *Cxcr4* and *Cxcl12* expression was observed in spinal cord of DA (*RT1^av1^*) rats immunized with CFA and MOG 1-125 in CFA (ANOVA, *P<0.001). There was an upregulation of *Cxcr4* and *Cxcl12* mRNA in DA (*RT1^av1^*) rats immunized with MOG 1-125 in CFA compared to DA (*RT1^av1^*) rats immunized with CFA alone (ANOVA, *P<0.01). Quantitative PCR results are expressed as 2^−ΔΔCT^ values. Numbers are mean±s.e.m.

Next, we assessed the mRNA expression of *Cxcr4* (Fig. 2A) and its ligand *Cxcl12* (Fig. 2B) in spinal cord of either naive DA (*RT1^av1^*) rats or DA (*RT1^av1^*) rats immunized with IFA or CFA alone or MOG 1-125 in IFA or MOG 1-125 in CFA (each *n*=4). Upregulation of both *Cxcl12* and *Cxcr4* mRNA expression was observed in CFA- and MOG 1-125 in CFA-immunized DA (*RT1^av1^*) rats in spinal cord compared to naive rats, IFA-injected or MOG 1-125 in IFA-immunized DA (*RT1^av1^*) rats (ANOVA, *P*<0.001). Increased *Cxcl12* and *Cxcr4* mRNA expression was observed in MOG 1-125 in CFA- compared to CFA-immunized DA (*RT1^av1^*) rats (ANOVA, *P*<0.01).

### *Cxcr4* and CXCL12 in individuals with MS

**Fig. 3.**
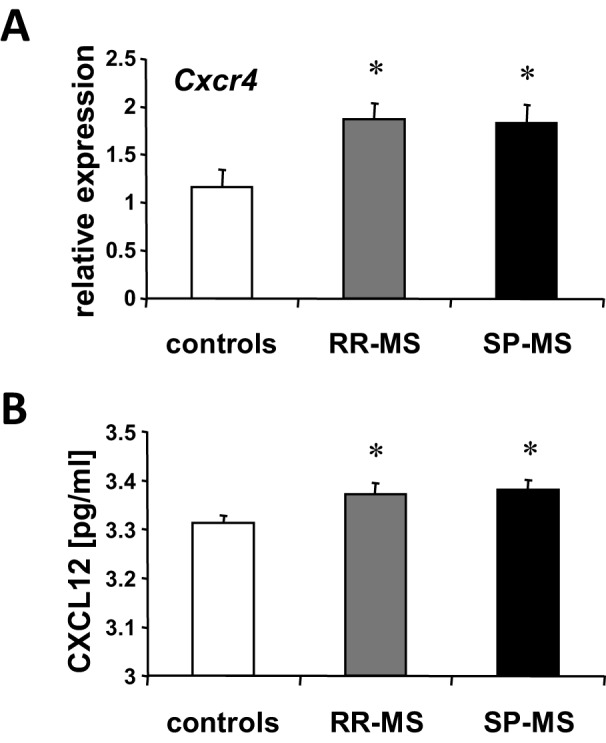
***Cxcr4* expression in white blood cells and CXCL12 protein in serum of individuals with MS.** (A) *Cxcr4* mRNA was quantified by real-time PCR from white blood cells of individuals with relapsing-remitting MS (RR-MS; gray bars, *n*=32), secondary chronic-progressive MS (SP-MS; black bars, *n*=22) and controls (white bars, *n*=25). Upregulation of *Cxcr4* in both patient groups compared to controls was observed (ANOVA, *P<0.05). (B) CXCL12 serum levels were assessed by ELISA in individuals with RR-MS (gray bars, *n*=24), SP-MS (black bars, *n*=28) and controls (white bars, *n*=21). Increased serum levels of CXCL12 were measured in both MS groups compared to controls (ANOVA, *P<0.05). Quantitative PCR results are expressed as 2^−ΔΔCT^ values. Numbers are mean±s.e.m.

Upregulation of *CXCR4* mRNA was also observed in white blood cells of individuals with MS with a relapsing-remitting disease course (RRMS; *n*=32) and a secondary chronic progressive disease course (SPMS; *n*=22), and compared to controls (*n*=25, ANOVA, RRMS and SPMS each *P*<0.05) (Fig. 3A, Tables S6 and S7). Also, we detected increased protein CXCL12 serum levels in both individuals with RRMS (*n*=24) and SPMS (*n*=28) compared to controls (*n*=21, ANOVA, RRMS and SPMS each *P*<0.05) (Fig. 3B, Tables S6 and S7).

### EAE in B6.129P2-*Cd38^tm1Lnd^*/J mice

**Fig. 4.**
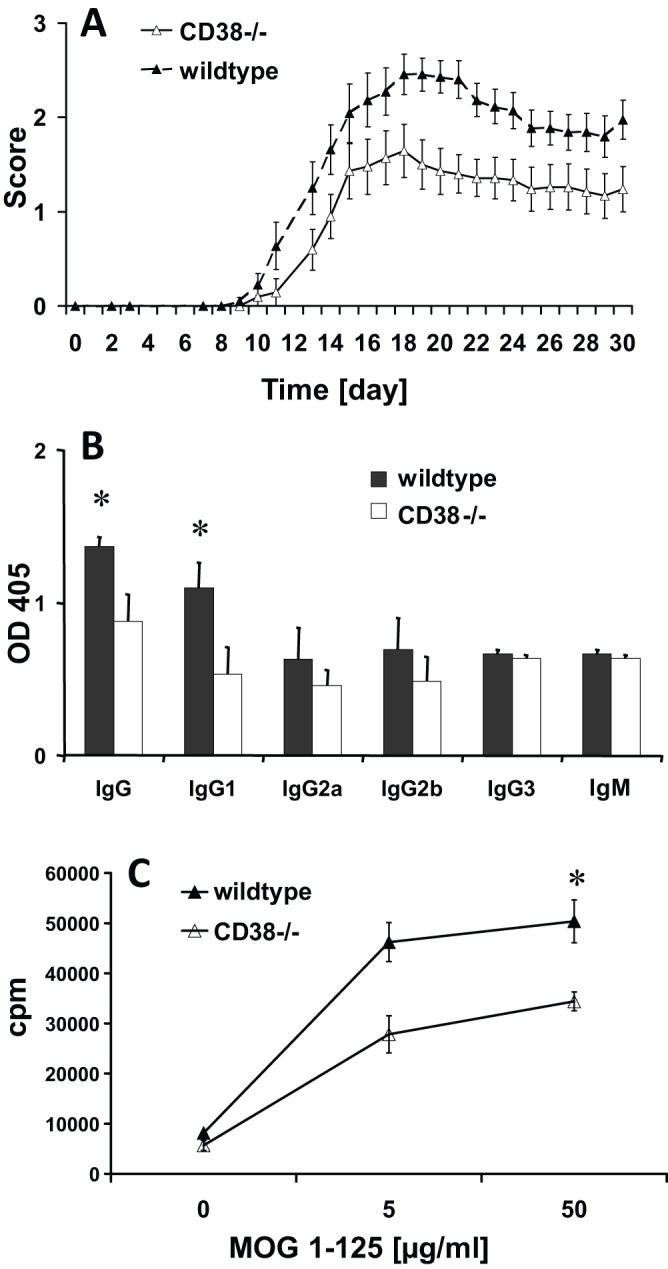
**EAE in B6.129P2-*Cd38^tm1Lnd^*/J mice.** (A) EAE was induced in female B6.129P2-*Cd38^tm1Lnd^*/J mice (white triangles) and C57BL/6J 000664 controls (black triangles) with MOG 1-125 in CFA. On days 0 and 2 p.i., mice received an intravenous injection of 150 ng pertussis toxin. EAE was scored as follows: 0, no disease; 1, tail paralysis; 2, paraparesis; 3, paraplegia; 4, tetraparalysis; 5, moribund or dead. Immunization with the extracellular domain of MOG 1-125 resulted in B6.129P2-*Cd38^tm1Lnd^*/J mice (*n*=22) with lower disease severity compared to C57BL/6J 000664 mice (*n*=21) (*t*-test, cumulative disease score, *P*=0.01). (B) Antibodies against MOG 1-125 were measured by ELISA as described (Weissert et al., 2001). B6.129P2-*Cd38^tm1Lnd^*/J mice (white bars, *n*=4) had reduced IgG and IgG1 antibodies compared to C57BL/6J 000664 mice (black bars, *n*=4) (ANOVA, *P<0.05) on day 12 p.i. (C) T-cell responses upon restimulation against MOG 1-125 from draining lymph nodes (LNs) on day 12 p.i. were reduced in B6.129P2-*Cd38^tm1Lnd^*/J (white triangles, *n*=4) compared to C57BL/6J 000664 (black triangles, *n*=4) mice (*t*-test for stimulation with 50 µg/ml MOG 1-125, *P=0.05). Numbers are mean±s.e.m.

*Cd38* was strongly upregulated in encephalitogenic LN cells. To functionally validate our data and to elucidate the role of CD38 in EAE, we induced disease with the extracellular domain of MOG (MOG 1-125) in *Cd38*-knockout (B6.129P2-*Cd38^tm1Lnd^*/J) mice and appropriate controls. We found reduced disease severity in MOG 1-125-immunized B6.129P2-*Cd38^tm1Lnd^*/J mice (*n*=22) compared to wild-type control mice (*n*=21, *t*-test, cumulative disease score *P*<0.01) (Fig. 4A). Next, we determined the height of the antibody response to MOG 1-125. Reduced anti-MOG IgG autoantibody responses in B6.129P2-*Cd38^tm1Lnd^*/J mice (*n*=4) compared to wild-type control mice (*n*=4, ANOVA, *P*<0.05) after immunization with MOG 1-125 were seen (Fig. 4B) on day 12 post-immunization (p.i.). Furthermore, also T-cell responses upon restimulation with MOG 1-125 were reduced in the B6.129P2-*Cd38^tm1Lnd^*/J mice (*n*=4) on day 12 p.i. compared to control mice (*n*=4, *t*-test for stimulation with 50 µg/ml MOG 1-125, *P*<0.05) as measured in a proliferation assay, indicating in addition a T-cell priming or expansion defect in B6.129P2-*Cd38^tm1Lnd^*/J mice (Fig. 4C).

### Targeting cellular migration by FTY720

**Fig. 5.**
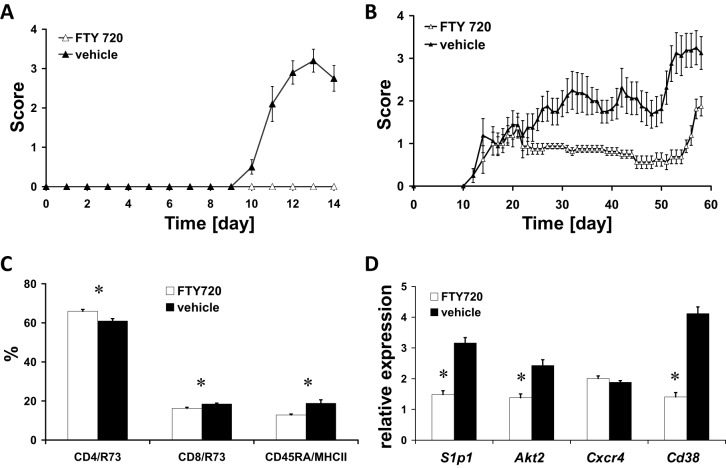
**Influence of FTY720 in EAE.** (A) Female LEW.1AV1 (*RT1^av1^*) rats were immunized with MOG 1-125 in CFA. EAE was scored as follows: 0, no disease; 1, tail paralysis; 2, paraparesis; 3, paraplegia; 4, tetraparalysis; 5, moribund or dead. FTY720 completely inhibited EAE in rats treated orally with 0.4 mg/kg body weight from day 0 p.i. daily (white triangles, *n*=10) as compared to vehicle (PBS)-treated controls (black triangles, *n*=10) (*t*-test, cumulative disease score, *P*<0.0001). (B) Female DA (*RT1^av1^*) rats treated orally with 0.4 mg/kg body weight FTY720 daily starting on day 21 p.i. after a first bout of disease (white triangles, *n*=8) showed a reduced disease course compared to vehicle (PBS)-treated controls (black triangles, *n*=8) (*t*-test, cumulative disease score day 21-44 p.i., *P*=0.01). On day 44 p.i., rats were boosted with MOG 1-125 in CFA. Both groups relapsed but with a better outcome for the FTY720-treated group (*t*-test, cumulative disease score day 45-56 p.i., *P*<0.0001). (C) FACS analysis of cells from draining lymph nodes (LNs) demonstrated an increase in relative size of the CD4 T-cell compartment and a decrease in the size of the CD8 and B-cell compartment in FTY720-treated (white bars, *n*=10) rats compared to controls (black bars, *n*=9) (ANOVA, *P<0.01) on day 14 p.i. (D) Expression of *S1p1* (*Edg1*), *Akt2*, *Cxcr4* and *Cd38* was assessed in FTY720-treated rats (white bars, *n*=9) compared to vehicle-treated controls (black bars, *n*=9) by quantitative PCR. FTY720 treatment led to downregulation of *S1p1* and *Akt2* as well as *Cd38*, but not of *Cxcr4* (ANOVA for all except *Cxcr4*, *P<0.0001) on day 14 p.i. from cells derived from LNs. Results are expressed as 2^−ΔΔCT^ values. Numbers are mean±s.e.m.

We found an upregulation of *Akt* in our differential gene expression studies (Table 1). FTY720 is an S1P-receptor modulator known to influence T-cell trafficking by an *Akt*-dependent mechanism, as does CXCR4 (Mandala et al., 2002; Cyster, 2005; Lee et al., 2001; Brinkmann et al., 2002; Matloubian et al., 2004). We evaluated inhibition of *Akt*-dependent cell trafficking in MOG 91-108-immunized LEW.1AV1 (*RT1^av1^*) rats. Disease was completely inhibited in rats treated from day 0 p.i. with FTY720 (*n*=10) as compared to the vehicle-treated controls (*n*=10, *t*-test, cumulative disease score *P*<0.0001) (Fig. 5A). Next, we tested the efficacy of FTY720 to treat established relapsing-remitting MOG 1-125-induced EAE in the DA (*RT1^av1^*) rat. FTY720 treatment was started on day 21 p.i., after the first bout of disease (*n*=8), and showed a significant effect on the disease course as compared to vehicle treatment (*n*=8, *t*-test, cumulative disease score day 21-44 p.i., *P*<0.01) (Fig. 5B). To further examine the beneficial effect of FTY720 treatment on EAE, we boosted the rats with MOG 1-125 on day 44 after the first immunization. Although a relapse was induced in both groups, the DA rats under FTY720 treatment had a better clinical outcome compared to the vehicle-treated animals (*t*-test, cumulative disease score day 45-56 p.i., *P*<0.0001). On day 14 p.i., FTY720 treatment led to an increase of the relative size of the CD4 T-cell compartment (ANOVA, *P*<0.01) in the treated LEW.1AV1 (*RT1^av1^*) rats (*n*=10) compared to controls (*n*=9) concurrent with a decrease in the CD8 T-cell (ANOVA, *P*<0.01) and the B-cell (ANOVA, *P*<0.01) compartment (Fig. 5C; Fig. S1). FTY720 treatment (*n*=9) compared to controls (*n*=9) led to the downregulation of mRNA expression of its receptor, *S1p1* (ANOVA, *P*<0.0001), and of *Akt2* (ANOVA, *P*<0.0001), one of the genes involved in the intracellular signaling cascade connected to S1P1 and CXCR4, on day 14 p.i. Treatment also had a negative effect on the expression levels of *Cd38* (ANOVA, *P*<0.0001). In contrast, the expression of *Cxcr4* was not altered (ANOVA, not significant) (Fig. 5D).

## DISCUSSION

In this study we identified gene networks that are crucially involved not only in raising an autoantigen-specific immune response but also in constituting encephalitogenicity. We analyzed the expression and functional relevance of genes and their products expressed on lymphocytes after immunization with the encephalitogenic MOG 91-108 peptide in adjuvant, the non-encephalitogenic MOG 73-90 peptide in adjuvant, adjuvant alone or naive rats. Most interestingly, in the comparison of MOG 91-108 in adjuvant to MOG 73-90 in adjuvant immunized rats, compared to the other analyses, only a small number of genes were differentially expressed. These genes seem to be of major importance because they are genes involved in the encephalitogenic response leading to disease manifestation. From the overall expression data, we selected three genes that were upregulated in many comparisons: *Cxcr4*, *Cd38* and *Akt*. We performed functional studies regarding these genes in EAE and analyzed tissue samples from individuals with MS.

CXCR4 (CD184) is a seven-transmembrane G-coupled receptor expressed by a number of tissues, including cells of the immune system (Campbell et al., 2003). *Cxcr4*-knockout mice die *in utero* or perinatally and do not only have defects in the hematopoietic system (impairment of myeloid and B-cell generation, reduced proliferation of triple-negative and double-positive lymphocytes), but also in the circulatory system and in the CNS (Zou et al., 1998; Tachibana et al., 1998). Overexpression of CXCR4 on T cells induces their accumulation in the bone marrow and reduction of these cells in the peripheral blood. CXCR4 signaling leads to a prolonged protein kinase B (AKT) and extracellular signal-regulated kinase 2 activation in T cells. AKT activation promotes cell survival and can act as a co-stimulation for T-cell activation (Tilton et al., 2000). CXCR4 has only one known cognate ligand, which is CXCL12. CXCL12 is constitutively produced by stromal and endothelial cells. CXCL12 activates numerous signaling pathways, such as receptor-associated trimeric G proteins, phospholipase Cγ, PI3K and small G proteins (Pawig et al., 2015). Signaling through these receptors leads to an increase in the intracellular calcium concentration, cytoskeleton reorganization and cellular migration. Several modulating factors such as phosphatases, regulator of G-protein signaling, adaptor proteins and ubiquitin may affect signaling and/or chemotactic response of CXCR4 to its ligand (CXCL12). An important function of CXCR4/CXCL12 is the regulation of bone-marrow homeostasis and lymphocyte trafficking. Chemotaxis and integrin-mediated adhesion are the main cellular responses to CXCL12. *Cxcl12*-knockout mice display the same phenotype as *Cxcr4*-knockout mice (Nagasawa et al., 1996).

In autoimmunity there are indications that the interaction of CXCR4 and CXCL12 could be important. CXCL12 recruits B cells to inflamed glomeruli, in which these cells can produce autoantibodies (Balabanian et al., 2003). Also, in rheumatoid arthritis, CXCR4 and CXCL12 have been proposed to be important in the disease precipitation (Zhang et al., 2005). A role of CXCR4 and CXCL12 in EAE (Meiron et al., 2008; Kohler et al., 2008; McCandless et al., 2006) as well as in MS (Azin et al., 2012; Krumbholz et al., 2006) has been described.

Our data indicate that, also in MOG-induced EAE and possibly in MS, the interaction of CXCR4 and CXCL12 is of paramount importance for disease development. We found specific upregulation of *Cxcr4* on cells derived from LNs and eluted from the CNS of rats immunized with encephalitogenic MOG 91-108 peptide in comparison to controls. In addition, we measured upregulation of *Cxcl12* and *Cxcr4* in spinal cords in EAE rats compared to controls. CXCL12 is upregulated in the CNS of individuals with MS (Krumbholz et al., 2006 and our own unpublished observations). Together, the presented data would argue for a scenario in which LN-derived cells are, in the context of an encounter with an encephalitogenic antigen, activated and migrate towards CXCL12 in the CNS. This is further underscored by the fact that nitric oxide enhances lipopolysaccharide (LPS)-induced expression of CXCR4 and migration towards CXCL12 (Giordano et al., 2006).

CD38 is a membrane-associated type II glycoprotein that acts both as a receptor and enzyme (Cockayne et al., 1998; Kato et al., 1999; Salmi and Jalkanen, 2005). As an enzyme it catalyzes NAD+ into cyclic ADP-ribose and further into ADP-ribose. It also regulates Ca^2+^ levels from ryanodine receptor stores. CD38 is expressed on a variety of myeloid and lymphoid cells. CD38 ligation in B cells leads to tyrosine phosphorylation of several intracellular proteins such as Syk, p85 of phophatidylinositol-3 kinase and phospholipase C-γ (Silvennoinen et al., 1996). CD38 ligation in T cells results in phosphorylation of the Raf-1/MAP kinase and CD3-ζ/ZAP-70 signaling pathway (Zubiaur et al., 1997). Furthermore, it is involved in dendritic cell migration and adhesion between lymphocytes and endothelial cells (Partida-Sanchez et al., 2004). B6.129P2-*Cd38^tm1Lnd^*/J mice show a slight reduction of antibody titers to T-cell-dependent antigens (Cockayne et al., 1998). Additionally, these mice had increased susceptibility to bacterial infections, which is thought to be caused by a defective chemotactic response of neutrophils towards bacteria, underscoring a role in innate immunity as well (Partida-Sanchez et al., 2001).

We induced EAE in *Cd38*-knockout mice (B6.129P2-*Cd38^tm1Lnd^*/J) and controls. *Cd38*-knockout mice had a reduced disease severity and lower autoantibody and T-cell responses as compared to the controls. These findings show the importance of CD38 in EAE and possibly MS. This work forms the basis for further analysis of the involved cellular compartments and regulation of human disease (Mayo et al., 2008; Lischke et al., 2013).

FTY720 is a potent drug that affects lymphocyte trafficking and homing. Our studies and studies by others (Brinkmann et al., 2002; Balatoni et al., 2007) showed a strong beneficial effect of FTY720 on EAE under various experimental settings. The drug was approved for treatment of MS (Kappos et al., 2010). We demonstrate that treatment of MOG-induced EAE with FTY720 impacts the gene expression of the genes that are involved in encephalitogenic immune responses. This further validates our approach. We measured changes in expression of *S1P1*, *Akt2* and *Cd38*. Interestingly, no changes in expression of *Cxcr4* was observed. The reason for this is not fully understood and deserves further investigations.

In conclusion, in this study we have identified genes that are involved not only in raising autoantigen-specific immune responses but which constitute encephalitogenicity. The immunization with encephalitogenic peptides induces a network of genes involved in activation and migration of lymphocytes. Based on this platform, we have established the paramount importance of G-coupled proteins in encephalitogenicity of adaptive immune responses. We speculate that similar involvement might operate also in other autoimmune diseases and possibly in transplant rejection, thereby establishing common mechanisms. These pathways might be valuable targets of therapeutic approaches as we have shown attenuation of EAE after treatment with FTY720 or reduced EAE severity in CD38-deficient mice. These findings not only validate our gene expression data, but also underscore the importance of the rat EAE model in translational medicine.

## MATERIALS AND METHODS

### Animals and EAE induction

Female rats or mice, 10-14 weeks of age, were used in all experiments. LEW.1AV1 (*RT1^av1^*) rats were obtained from Hans Hedrich (Central Animal Laboratory, Hannover Medical School, Hannover, Germany) and DA (*RT1^av1^*) rats were obtained from Harlan Winkelmann (Borchen, Germany). Female B6.129P2-*Cd38^tm1Lnd^*/J mice and the appropriate controls (C57BL/6J 000664) were purchased from the Jackson Laboratory (Bar Harbor, USA).

Animals were bred and kept under specific pathogen-free conditions. Animals were injected intradermally at the base of the tail (rats) or both flanks (mice) with 100 µg of MOG 91-108 (rats) or 50 µg of rat recombinant MOG 1-125 (rats) or 100 µg MOG 35-55 (mice) or 20 µg rat recombinant MOG 1-125 (mice). The antigens in a total volume of 100 µl were mixed with 100 µl of CFA (1:1). A total of 100 µl of CFA consisted of IFA (Sigma-Aldrich, St Louis, MO) and 500 µg for rats or 400 µg for mice of heat-inactivated *Mycobacterium tuberculosis* (strain H37 RA; Difco Laboratories, Detroit, MI) (Weissert, 2016). Mice additionally received 100 ng of pertussis toxin (Calbiochem, Darmstadt, Germany) on day 0 and 2 intravenously. Some groups of rats were also injected with 100 µl IFA mixed with 100 µl MOG 1-125 in PBS (50 µg) without the addition of *Mycobacterium tuberculosis* or with IFA or CFA alone. The clinical scoring was as follows: 0=no illness; 1=tail weakness or paralysis; 2=hind-leg paraparesis or hemiparesis; 3=hind-leg paralysis or hemiparalysis; 4=tetraparesis or moribund. All experiments were approved by the regional board in Tübingen, Germany.

### Human samples

Blood samples were obtained after consent from individuals with MS and controls. The characteristics of the MS individuals and controls are indicated in Tables S6 and S7. The research was approved by the Ethics Committee of the University of Tübingen in Germany (Permission 125/2001).

### Isolation of CNS-infiltrating cells

Infiltrating cells from the CNS were prepared as described before (Weissert et al., 1998a, 2001). In brief, rats were perfused with cold PBS, and brains and spinal cords were dissected out on day 12 p.i. Subsequently, brains and spinal cords were homogenized in 10 ml 50% Percoll/0.1% BSA/1% glucose (Amersham Pharmacia Biotech) containing 500 U DNase type I (Life Technologies). Ten ml of 50% Percoll was added to each sample after homogenization. A discontinuous Percoll gradient was obtained by adding 7 ml of 63% Percoll below and 20 ml of 30% Percoll above the sample. Samples were centrifuged for 40 min at 1000 ***g*** at 4°C. Lymphocytes were collected from the 63/50% Percoll interface. The cells were subsequently washed twice in 15-25 ml PBS with centrifugation at 600 ***g*** for 15 min at 4°C.

### Isolation of mononuclear cells from lymph nodes and spleens

Draining inguinal LNs and spleens were dissected out under deep anesthesia. LNs were disrupted and mononuclear cells (MNCs) washed twice in Dulbecco's modified Eagle's medium (DMEM; Life Technologies, Paisley, UK), resuspended in complete medium (CM) containing DMEM supplemented with 5% fetal calf serum (PAA Laboratories Linz, Austria), 1% penicillin/streptomycin (Life Technologies), 1% glutamine (Life Technologies) and 50 µM 2-mercaptoethanol (Life Technologies), and flushed through a 70-µm plastic strainer (Falcon; BD Biosciences, Franklin Lakes, NJ). MNCs from spleen were prepared in the same way as from LNs with the difference that red blood cells were lysed with lysis buffer consisting of 0.15 M NH_4_Cl, 10 mM KHCO_3_ and 0.1 mM Na_2_ EDTA adjusted to pH 7.4. CD4^+^ cells were isolated by anti-rat CD4 microbeads using MACS technology (Miltenyi Biotech, Bergisch Gladbach, Germany) according to the manufacturer's instruction.

### CD4^+^ cell purification

CD4^+^ cells from the LNs were purified by MACS (Miltenyi Biotech, Bergisch Gladbach, Germany) and subsequently analyzed by Affymetrix gene array for differential gene expression.

### Spinal cord tissue

From PBS-perfused naive rats or in DA rats immunized with either IFA, CFA, MOG 1-125 in IFA or MOG 1-125 in CFA, spinal cord tissue was dissected and homogenized and subsequently assessed for mRNA expression.

### RNA preparation

Total RNA of brain-infiltrating leukocytes or lymphocytes or spinal cord tissue was isolated by using an RNeasy kit (Qiagen, Hilden, Germany) according to the manufacturer's instructions. The RNA quality was analyzed with a Bioanalyser 2100 (Agilent, Palo Alto, CA).

### Microarrays

Affymetrix microarrays of the type RG U34 A (Affymetrix Inc., Santa Clara, CA) representing approximately 7000 full-length genes and 1000 expressed sequence tag (EST) clusters were used. For the purified CD4^+^ cells, the rat expression set 230A (Affymetrix) containing about 30,000 features was used. For each array, samples from at least three rats were pooled. Biotin-labeled cRNA was prepared and hybridized to the arrays. In brief, double-stranded cDNA was synthesized from whole RNA using a superscript choice kit (Invitrogen) with a T7-(dT)24 primer (Metabion) and *in vitro* transcribed into biotin-labeled cRNA. After hybridization, gene arrays were washed and stained by a fluidics station (Affymetrix) and scanned by a confocal laser scanning microscope (Agilent).

The data were analyzed using the microarray suite software, micro DB, and data-mining tool (Affymetrix). Only genes and ESTs that were ‘present’ and gave a difference call of either ‘increase’ or ‘decrease’ according to the Affymetrix software were included in further analysis. The extent of differential expression is expressed as an SLR. For the tables, the cut-off was set to an SLR of 1 signifying a twofold change in expression. Doubles, ESTs and sequences not corresponding to a gene were not included in the tables.

### Real-time PCR

To avoid amplification/detection of contaminating genomic DNA, extracted RNA was treated with RNase free DNase (Promega, Madison, WI). Subsequently, cDNA was synthesized by reverse transcription with Moloney murine leukemia virus reverse transcriptase and random pdN6 primers in the presence of RNase inhibitor (Promega). Amplification was performed on an Applied Biosystems Prism 7000 Sequence Detection System (Applied Biosystems, Foster City, CA) using a SYBR green protocol. Results were expressed as 2^−ΔΔCT^ values. The primer sequences 5′ to 3′ were as follows:

#### Rat primers

*Gapdh*: forward: GGTTGTCTCCTGTGACTTCAA
  reverse: CATACCAGGAAATGAGCTTCAC
*S1p1*: forward: TAGCCGCAGCAAATCAGAC
  reverse: GCAGCAGTGGAGAAAGAGAGA
*Cxcr4*: forward: GATGGTGGTGTTCCAGTTCC
  reverse: CAGCTTGGAGATGATGATGC
*Cxcl12*: forward: CGATTCTTTGAGAGCCATGT
  reverse: AGGGCACAGTTTGGAGTGTT
*Cd38*: forward: AGGACACACTGCTGGGCTAT
  reverse: CAGGGTTGTTGGGACAATTT
*Akt2*: forward: GAAGACTGAGAGGCCACGAC
  reverse: GGGAGCCACACTTGTAATCC

#### Human primers

*h18s*: forward: CGGCTACCACATCCAAGGAA
  reverse: GCTGGAATTACCGCGGCT
*Cxcr4*: forward: CATCAGTCTGGACCGCTACC
  reverse: GGATCCAGACGCCAACATAG.

### FACS

FITC-conjugated monoclonal antibody (mAb) against CD45RA (OX-33) and TCRAB (R73) and phycoerythrin (PE)-conjugated mAb against CD4 (OX-35) and MHC II (OX-6) and appropriate isotype controls were purchased from Becton Dickinson (Heidelberg, Germany). Flow cytometry was performed on a FACScalibur running with Cellquest software (Becton Dickinson). Cells were gated on the lymphocyte population in the forward scatter (FSC)-side scatter (SSC) dot plot. For data analysis, Flowing Software 2.5.1 (Turku Center for Biotechnology, Finland) was used.

### ELISA

Serum taken at the time point of euthanasia was subject to an anti-MOG autoantibody ELISA. ELISA plates (96-well; Nunc, Roskilde, Denmark) were coated with 2.5 µg/ml (100 µl/well) MOG 1-125 overnight at 4°C. Plates were washed with PBS/0.05% Tween 20 and blocked with milk powder for 1 h at room temperature. After washing, diluted serum samples were added and plates were incubated for 1 h at room temperature. Then, plates were washed and rabbit anti-mouse antiserum (IgG, IgG1, IgG2a, IgG2b, IgG3, IgM; Nordic, Tilburg, The Netherlands) was added and incubated for 1 h at room temperature. Plates were washed prior to the addition of peroxidase-conjugated goat anti-rabbit antiserum (Nordic) diluted in PBS/0.05% Tween 20. After 30 min incubation, plates were washed and bound antibodies were visualized by addition of 2,2′azino-bis(3-ethylbenzothiazoline-6-sulphonic acid) (ABTS) (Roche Diagnostics, Mannheim, Germany). After 15 min of incubation, optical density was read at 405 nm.

ELISA with human serum was performed according to the instructions of the manufacturer [Quantikine human SDF1alpha Immunoassay (R&D Systems, Minneapolis, MN)].

### Proliferation assay

Cells from draining LNs were prepared as described above and cultured in the presence of rrMOG in 96-well plates as described by Weissert et al. (1998b). Cells were cultured for 48 h and pulsed with 1 µCi [^3^H]thymidine for the last 18 h. The incorporation of [^3^H]thymidine was measured using a beta-scintillation counter.

### FTY720 treatment

Rats were treated with FTY720 (generous gift of Novartis AG, Switzerland) and received a daily dose of 0.4 mg/kg body weight in sterile water by oral gavage as described (Balatoni et al., 2007).
